# Verification of the Efficacy of Mexiletine Treatment for the A1656D Mutation on Downgrading Reentrant Tachycardia Using a 3D Cardiac Electrophysiological Model

**DOI:** 10.3390/bioengineering9100531

**Published:** 2022-10-07

**Authors:** Ali Ikhsanul Qauli, Yedam Yoo, Aroli Marcellinus, Ki Moo Lim

**Affiliations:** 1 Department of IT Convergence Engineering, Kumoh National Institute of Technology, Gumi 39177, Korea; 2 Robotics and Artificial Intelligence Engineering, Faculty of Advanced Technology and Multidiscipline, Universitas Airlangga, Surabaya 60115, Jawa Timur, Indonesia; 3 Department of Medical IT Convergence Engineering, Kumoh National Institute of Technology, Gumi 39253, Korea

**Keywords:** LQT3, mutation, reentry, APD, alternant

## Abstract

The SCN5A mutations have been long associated with long QT variant 3 (LQT3). Recent experimental and computation studies have reported that mexiletine effectively treats LQT3 patients associated with the A1656D mutation. However, they have primarily focused on cellular level evaluations and have only looked at the effects of mexiletine on action potential duration (APD) or QT interval reduction. We further investigated mexiletine’s effects on cardiac cells through simulations of single-cell (behavior of alternant occurrence) and 3D (with and without mexiletine). We discovered that mexiletine could shorten the cell’s APD and change the alternant’s occurrence to a shorter basic cycle length (BCL) between 350 and 420 ms. The alternant also appeared at a normal heart rate under the A1656D mutation. Furthermore, the 3D ventricle simulations revealed that mexiletine could reduce the likelihood of a greater spiral wave breakup in the A1656D mutant condition by minimizing the appearance of rotors. In conclusion, we found that mexiletine could provide extra safety features during therapy for LQT3 patients because it can change the alternant occurrence from a normal to a faster heart rate, and it reduces the chance of a spiral wave breakup. Therefore, these findings emphasize the promising efficacy of mexiletine in treating LQT3 patients under the A1656D mutation.

## 1. Introduction

Until now, cardiac arrhythmia has been recognized as a global health problem [1]. Sudden cardiac death (SCD) is responsible for 250,000–300,000 fatalities in the United States annually, according to [2]. They also expected that in industrialized nations, an annual incidence of 50–100/100,000 people would acquire SCD. In 2018, one study found that the incidence of fatal cardiovascular disease (CVD) in Asians was 3.68 (95 percent confidence interval: 2.84–4.53) events per 1000 person-years [3]. Long QT syndrome (LQTS), caused by a mutation in an ion channel, is one of the leading causes of inherited CVD and SCD [4]. Previous research [5] also found a 1:2000 chance of developing a genetic disease among healthy live babies.

SCN5A mutations have been linked to LQTS 3, which codes for the α-subunit of the Na+ channel (Nav1.5), according to [6]. A review report [7] found that the LQT3–caused mutations in SCN5A extend the QT interval by minor net increases in the INa current. These are frequently due to an aberrant persistent or sustained late sodium current or to defective a Nav1.5 inactivation observed across the full voltage range and time-course-of-action potential plateau that disrupts the delicate balance between inward and outward currents [8,9]. Furthermore, the QT interval of LQT3 patients is significantly prolonged, especially during rest [10,11], and shortened during a fast heart rate. It has long been hypothesized [12]—and recently proved in a murine model [13]—that diurnal variation in cardiac repolarization patterns may be responsible for the temporally dependent vulnerability to ventricular arrhythmias seen in LQT3 and other heritable cardiac arrhythmia syndromes. However, the precise electrophysiological mechanism underlying this phenomenon is still not fully understood.

In a newborn baby with QT prolongation and non-sustained ventricular tachycardia (NSVT), a unique SCN5A variation known as A1656D was recently discovered [14]. Kim et al. reported that under the influence of mexiletine, A1656D exhibited a distinct response that resulted in the suppression of NSVT and the appearance of premature atrial contraction. Furthermore, another study [15] found that sodium channel mutations N1325S and R1623Q had an unusual reaction to mexiletine, where they could completely restore the electrocardiogram (ECG) and eliminate arrhythmias.

As the sodium blocker, mexiletine has been known to selectively inhibit the slowly–inactivating component of the inward tetrodotoxin–sensitive sodium current $\left(I_{\mathrm{NaL}}\right)$ without affecting the speed of action potential upstroke [16,17]. The mexiletine can also abbreviate the QT interval shown on ECG without widening the QRS duration [16,18]. In LQT3 patients, the $I_{\mathrm{NaL}}$ is greatly enhanced in a slower heart rate because of a slower inactivation of the $I_{\mathrm{NaL}}$ in bradycardia conditions [19]. Some researchers found that a faster heart rate can reduce the $I_{\mathrm{NaL}}$, shorten APD and QT interval, thus diminishing the $I_{\mathrm{NaL}}$ becomes the basis for LQT3 treatment [20,21].

However, some questions remain about the sodium blocker’s effectiveness in LQT3 patients. The significance of sodium channel blockers for LQT3 individuals has been mixed in several trials [22,23]. In this context, a better knowledge of the mechanism of sodium blockers for treating LQT3 patients has become a top goal. The efficacy of mexiletine for treating LQT3 patients has recently been demonstrated in a previous study [14,23,24]. Despite the extensive experimental evaluation of mexiletine for treating LQT3 patients, their findings require more investigation into the effects of mexiletine on a 3D ventricular model of the heart. The single-cell experimental studies may not anticipate additional impacts that a computer simulation at the single-cell level and a three-dimensional model can disclose.

Some researchers have adapted the combined experimental work and in silico simulation to assess the ion channel mutation. A study by Yu et al. [25] assessed the arrhythmia mechanism of the infarcted ventricles following remuscularization with pluripotent stem cell-derived cardiomyocytes (PSC-CMs). They concluded that the main ventricular tachycardia (VT) mechanism involved in patch remuscularization would be reentrant VT. Furthermore, Paci et al. [26] emphasized the ability of populations of in silico simulation to describe the experimental findings of the prolonged action potential as a product of LQT3 hiPSC-CMs mutation and predict differences in drug response of the ion channel mutation. Deo et al. [27] combined an experimental study of the mutation (E299V) in the KCNJ2 gene that encodes the strong inward rectifier K+ channel protein (Kir2.1) and 3D computational modeling of the heart incorporating the His-Purkinje network to predict the 20% reduction of sodium current that can cause reduction of ventricular excitability and increase the vulnerability of ventricular tachyarrhythmia. Kim et al. [14] also produced a computational model of the A1656D mutation, which demonstrated how various medications affect the AP shape of the ventricular cell. One of their significant discoveries is that mexiletine can reduce the APD of the A1656D mutation by restoring late sodium current inactivation kinetics.

The effects of mexiletine on A1656D mutations on single-cell ventricular cells (myocardial, endocardial, and epicardial) were further computationally elaborated in this paper. The study was also extended to a 3D ventricular model. We focused on reentrant arrhythmias that might arise due to alternants in single-cell simulation [28] and spiral waves in a 3D ventricular model.

## 2. Materials and Methods

**Figure 1:**
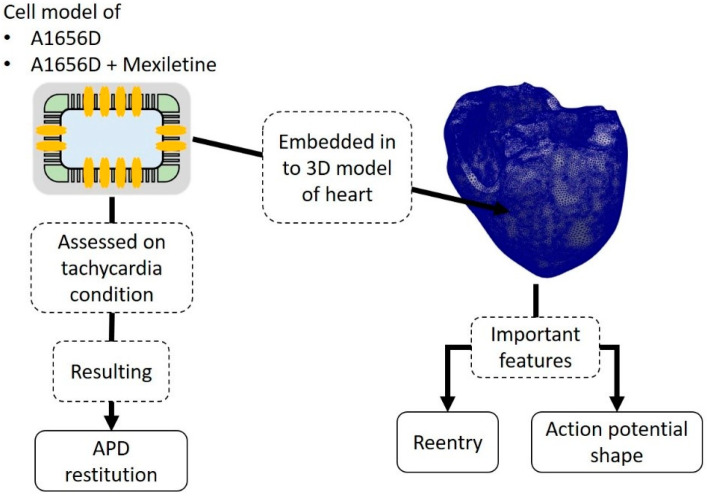
Diagram of the simulation protocol of single cell and 3D model of the heart.

In this work, the cell model incorporated in simulations was initially based on the ventricular cell model proposed by [29] with some modifications to resemble the A1656D mutation and the A1656D mutation under mexiletine as proposed by [14]. As shown in Figure 1, the main simulations consisted of single-cell simulation and 3D ventricular simulation. The single-cell simulation was used to study the tachycardia condition by the APD restitution graph showing the BCL region with alternant. Furthermore, the cell models were embedded into the 3D model of the ventricle so that it could allow us to observe the reentry and compare the shape of action potential among different conditions.

### 2.1. Model of Ventricular Cell under A1656D Mutation with Incorporated Drug Effects

A recent study by Kim et al. [14] examined and simulated a myocardial cell model based on the work of [29]. The differential equation that characterized the cell is as follows:

(1) $$C_{m}\frac{{\mathrm{dV}}_{m}}{\mathrm{dt}}=-\left(I_{\mathrm{ion}}+I_{\mathrm{stim}}\right),$$

where $C_{m}$ is the total membrane capacitance, $I_{\mathrm{stim}}$ is the stimulus current, and $I_{\mathrm{ion}}$ is the sum of ionic transmembrane currents. In addition, ten Tusscher et al. [30] proposed that the transmembrane currents consist of sodium current $\left(I_{\mathrm{Na}}\right)$, inward rectifier potassium current $\left(I_{K1}\right)$, transient outward potassium current $\left(I_{\mathrm{to}}\right)$, rapid delayed rectifier potassium current $\left(I_{\mathrm{Kr}}\right)$, slow delayed rectifier potassium current $\left(I_{\mathrm{Ks}}\right)$, L-type calcium current $\left(I_{\mathrm{CaL}}\right)$, sodium/calcium exchange current $\left(I_{\mathrm{NaCa}}\right)$, pump current $\left(I_{\mathrm{NaK}}\right)$, plateau Ca^2+^ current $\left(I_{\mathrm{pCa}}\right)$, plateau K^+^ current $\left(I_{\mathrm{pK}}\right)$, Ca^2+^ background current $\left(I_{\mathrm{bCa}}\right)$, and K^+^ background current $\left(I_{\mathrm{bNa}}\right)$ [29].

Furthermore, Kim et al. [14] proposed some changes to the $\left(I_{\mathrm{Na}}\right)$ modeling that alters the channel’s activation (m) and inactivation (h) information. Fast $\left(h_{f}\right)$ and slow $\left(h_{s}\right)$ kinetics were used to explain the h-gate [14]. The sodium current can be stated in the following way:

(2) $$m_{\infty }=\sqrt[{3}]{\frac{1}{1+e^{\left(V-V_{h\left(m_{\infty }\right)}\right)/k_{m}}}},$$

(3) $$m_{\tau }=\frac{a_{m}}{e^{b_{m}\left(V-V_{h_{m_{\tau }}}\right)/\mathrm{RT}}+e^{-c_{m}\left(V-V_{h_{m_{\tau }}}\right)/\mathrm{RT}}}+d_{m},$$

(4) $$h_{f_{\infty }}=h_{s_{\infty }}=\frac{1}{1+e^{\left(V-V_{h\left(h_{\infty }\right)}\right)/k_{h}}},$$

(5) $$h_{f_{\tau }}=\frac{a_{h_{f}}}{e^{b_{h_{f}}\left(V-V_{h_{f_{\tau }}}\right)/\mathrm{RT}}+e^{-c_{h_{f}}\left(V-V_{h_{f_{\tau }}}\right)/\mathrm{RT}}}+d_{h_{f}},$$

(6) $$h_{s_{\tau }}=\frac{a_{h_{s}}}{e^{b_{h_{s}}\left(V-V_{h_{s_{\tau }}}\right)/\mathrm{RT}}+e^{-c_{h_{s}}\left(V-V_{h_{s_{\tau }}}\right)/\mathrm{RT}}}+d_{h_{s}},$$

where $m_{\infty }$ is the steady-state activation, $h_{\infty }$ is the steady-state inactivation, V is the membrane potential (millivolt), R is the gas constant $\left(8.31{\;j\;\mathrm{mol}}^{-1}K^{-1}\right)$, T is the absolute temperature in Kelvin. The forward (α) and backward (β) rate constants of each gate can be calculated as follows:

(7) $$\alpha_{m}=\frac{m}{\tau_{m}},$$

(8) $$\beta_{m}=\frac{1-m}{\tau_{m}},$$

(9) $$\alpha_{h_{f}}=\frac{m_{f}}{\tau_{h_{f}}},$$

(10) $$\beta_{h_{f}}=\frac{1-h_{f}}{\tau_{h_{f}}},$$

(11) $$\alpha_{h_{s}}=\frac{m_{s}}{\tau_{h_{s}}},$$

(12) $$\beta_{h_{s}}=\frac{1-h_{s}}{\tau_{h_{s}}},$$

the relative contribution of fast and slow groups of h-gate to total open probability was $r_{f}$ and $r_{s}$ $\left(=1-r_{f}\right)$. Finally, the sodium current was calculated using the following formula:

(13) $$I_{\mathrm{Na}}=G_{{\mathrm{Na}}_{\max}}\times m^{3}\times \left(r_{f}\times h_{f}+r_{s}\times h_{s}\right)\times \left(V-E_{\mathrm{rev}}\right),$$

where $G_{\mathrm{Na}}$ is the maximum sodium channel conductance assumed to be 1125.0 for WT, 213.66 for A1656D, and 231.18 for A1656D under mexiletine. Furthermore, Tables 1–4 in [14] provide detailed values for the parameter set of m, $h_{f}$, and $h_{s}$ gates in WT, the A1656D mutation, and the A1656D mutation under mexiletine. Other associated constants for the $I_{\mathrm{Na}}$ modeling can be found in [14] and references therein.

For 3D ventricular simulation, by ignoring microscopic cardiac cell structure, the cardiac cells can be modeled as follows [30]:

(14) $$C_{m}\frac{\mathrm{dV}}{\mathrm{dt}}=-\left(I_{\mathrm{ion}}+I_{\mathrm{stim}}\right)+\frac{1}{\rho_{x}S_{x}}\frac{\partial^{2}V}{\partial x^{2}}+\frac{1}{\rho_{y}S_{y}}\frac{\partial^{2}V}{\partial y^{2}}+\frac{1}{\rho_{z}S_{z}}\frac{\partial^{2}V}{\partial z^{2}},$$

where $\rho_{x}$, $\rho_{y}$, and $\rho_{z}$ are the cell’s resistivity in x, y, and z directions; and $S_{x}$, $S_{y}$, and $S_{z}$ are the surface-to-volume ratio in x, y, and z directions, respectively. The 3D mesh incorporated in the electrical simulation consisted of 241,725 nodes and 1,298,751 tetrahedral elements.

### 2.2. Simulation Protocol

There were three primary simulations in the single-cell electrical simulation: wildtype (WT), the A1656D mutant, and A1656D under mexiletine. The tachycardia state was analyzed using an APD restitution (APDR) graph by varying the BCL from 1400 ms to 300 ms with a step of 10 ms and 20 pacings for each BCL. We applied current stimulation for every pace with a stimulus duration of 1.0 ms and stimulus amplitude of 52 pA/pF.

The A1656D mutant and A1656D under mexiletine were the two primary sections of the 3D ventricular simulations. The BCL of 1000 ms was used in all scenarios. Furthermore, we used the S1 protocol to execute a homogenous simulation in which we stimulated a node from the apex of the heart five times. With the standard S2 procedure, we additionally included reentry/arrhythmia simulation. The electrical stimulation was administered to the heart’s apex five times, and half of the stimulated nodes were reset right before the fifth planar wavefront. The stimulus might result in the reentry spreading to the resting nodes. Each 3D simulation time was set to 10 s for both the A1656D mutation and the A1656D mutation with mexiletine.

## 3. Results

**Figure 2:**
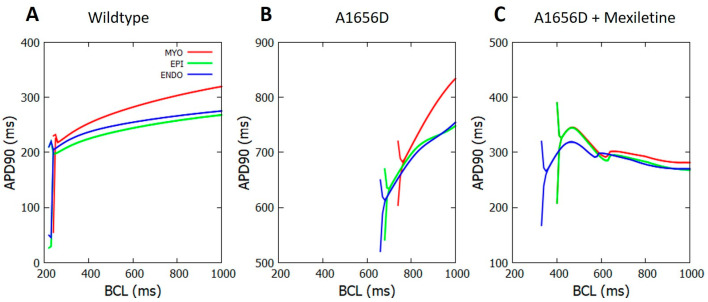
The APD as a function of BCL for WT, the A1656D mutant and A1656D under mexiletine.

Figure 2 depicts the APDR graph produced by a single-cell simulation with varied BCL values. The branches resulted in Figure 2 represent the alternants. The alternant occurs approximately at BCL between 240–260 ms for the WT in panel A. Among them, the myocardial cell-type results in the alternant with a relatively longer BCL of around 260 ms compared to both epicardial and endocardial cells that result in the alternant at BCL of approximately 240 ms. Furthermore, we can clearly see in panel B for A1656D mutation simulation that the A1656D mutation can cause APD prolongation and a shift of the occurrence of alternant from short BCL to longer BCL compared to WT of around 760 ms for myocardial cell-type, 700 ms for epicardial cell, and 680 ms for endocardial cell. On panel C, we can observe the effect of mexiletine on the A1656D mutation. Under mexiletine, myocardial and epicardial cell types converge to the same BCL of roughly 420 ms for the alternant to develop. However, with a shorter BCL of approximately 350 ms, the endocardial cell-type results in the alternant. We could also observe the progression of the APD values when the alternant occurs among all cell types from panels A to C. The difference in the APD values in WT is more negligible than in A1656D and A1656D under mexiletine. The highest difference in the APD values is observed between the myocardial–epicardial and endocardial cells at around 70 ms in panel C. 

**Figure 3:**
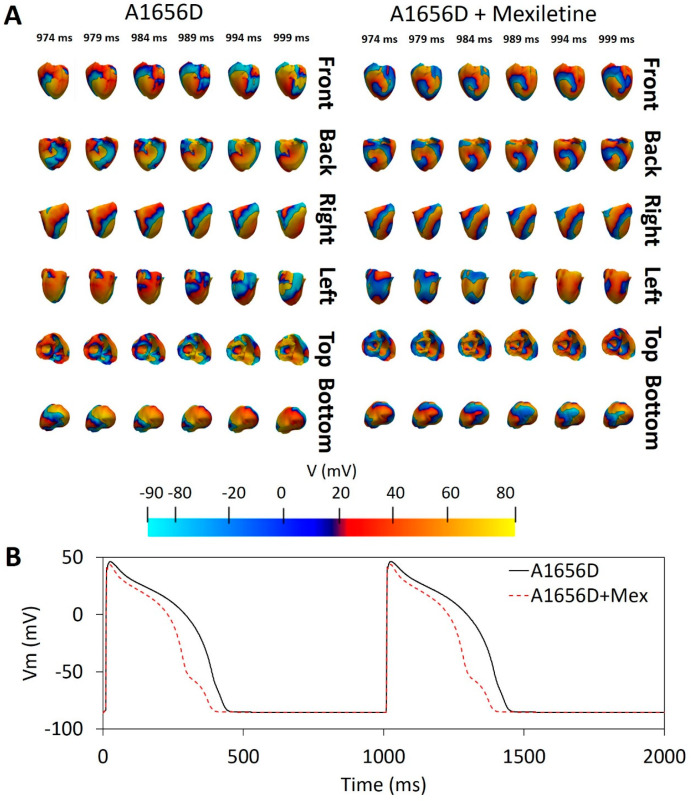
The outcomes of a 3D ventricular simulation. Panel (**A**) depicts the reentry dynamic from various angles, and panel (**B**) defines the action potential shape for A1656D and A1656D under mexiletine. The black star in Panel (**A**) shows the rotor. The cell type depicted in this illustration is the epicardia cell.

Figure 3 depicts the results of a 3D ventricular simulation. In panel A, we can see a comparison of electrical activity from the A1656D mutation and A1656D when treated with mexiletine. At 989 ms, we labeled the rotors formed upon reentry with a black star. We can see that the A1656D mutation has around five rotors in total. They are visible from the “front”, “back”, “left”, and “top” sides of the ventricular mesh. The A1656D mutation, on the other hand, has fewer rotors, with only four, one at the “front”, one at the “back”, and two at the “top” of the mesh model. The conduction velocity (CV) detected from the simulation was 19 cm/s for A1656D mutation, and 17.3 cm/s for A1656D mutation under mexiletine. Panel B compares the action potential shape of the epicardial cells with the effect of A1656D mutation and A1656D under mexiletine. The A1656D mutation resulted in a more prolonged APD as compared to the A1656D mutation when mexiletine was used.

## 4. Discussion

Figure 2 shows that three conditions (WT, A1656D mutation, and A1656D under mexiletine) can produce different APD profiles in the cell. In panel A, the three kinds of cells make APDs greater than 200 ms for instances before the alternant. In panel B, the APD of the cells varies significantly, with the shortest occurring more than 600 ms before the endocardial cell alternant. In panel C, mexiletine can shorten the APD from the A1656D mutation by more than 250 ms before the endocardial cell alternant. The APD shortening by mexiletine demonstrated in our data is consistent with earlier work indicating that the late sodium channel alteration generated by the A1656D mutation is controlled by mexiletine (known to be a late sodium blocker [16,17]). The blockage of late sodium channels can induce repolarization to occur faster, lowering APD. In the model used in this work, the late sodium current is represented by slow kinetics in the formulation of the $I_{\mathrm{Na}}$ as proposed by [14].

A high APD difference between two successive action potentials has a high chance of causing discordant alternants [31]. The high APD difference (indicated by the difference between the branch line) shown in Figure 2, panels B and C, may inform us that A1656D and A1656D under mexiletine could cause a discordant alternant more strongly than WT. However, as shown in Figure 2, the A1656D mutation under mexiletine exhibits a change in the alternant incidence towards shorter BCL or a higher heart rate compared to the A1656D mutation. Figure 2, panel B, shows that the alternant occurs between 680 and 760 ms of BCL in the A1656D mutation. The typical adult human heart rate is between 600 ms and 1000 ms of BCL [32], implying that the A1656D mutation might raise the risk of alternant within the normal heart rhythm. Meanwhile, under mexiletine conditions, the alternant occurs at around 350 ms to 420 ms of BCL for the A1656D mutation. Instead, the alternant might appear at a quicker heart rate of 350 ms BCL for endocardial cells and 420 ms BCL for epicardial and myocardial cells. Therefore, mexiletine–treated A1656D show a safer result by lowering the possibility of the alternant occurring at a normal heart rate compared to the pure A1656D mutation. Our findings are consistent with previous research [27], which also reported the occurrence of QT prolongation during rest condition of LQT3 patients. In addition, a recent study indicated that a higher heart rate might reduce the $I_{\mathrm{NaL}}$ and shorten APD [33]. As a result, the reduction in the $I_{\mathrm{NaL}}$ paired with the decline in APD substantially demonstrates the effectiveness of mexiletine in lowering the risk of the alternant in LQT3 patients during therapy. In fact, the $I_{\mathrm{NaL}}$ lowering has been utilized as the foundation of LQT3 therapy using pacemakers [20,21].

The results of the 3D ventricular simulation, as shown in Figure 3, reveal that mexiletine can reduce the number of rotors (from four to two) in the A1656D mutant scenario. More rotors in the A1656D mutant condition may result in greater chaotic electrical activity, which may cause more spiral wave breakup and ventricular fibrillation. According to [34], cardiac electrical restitution properties influence the breakup of reentrant wavefronts during cardiac fibrillation, known as the concordant alternant. The wavelength of the successive wave oscillates between short and long during the concordant alternant. Furthermore, [35] found that rotors may trigger the ventricles at such high frequencies that the wavefronts propagating from it break apart across a wide range of distances, resulting in ventricular fibrillation. As a result, the reduced number of rotors caused by mexiletine therapy for the A1656D mutation may reflect its efficacy in decreasing the risk of spiral wave breakup, which can lead to ventricular fibrillation.

We have limited our 3D ventricular reentry simulation analysis to the epicardial cell type only. Results may be different when simulated using other cell types. However, recent studies [36,37,38] showed that the epicardial could exhibit more alternants than other regions (myocardial and endocardial), which may be related to the buildup of fat on the epicardial surface. Therefore, we may assume that the results from the epicardial cell are sufficient to describe the occurrence of a spiral breakup. 

Furthermore, incorporating the iPSC-CMs into the existing simulation protocol may be beneficial for further study as iPSC-CMs could also mimic the specific genetic description of the LQT3 patients. However, the limitations of iPSC-CMs need to be considered. Cardiomyocytes generated from human iPSCs lack T-tubules and exhibit poor colocalization of calcium channels and ryanodine receptors [39]. As a result, special care should be taken when modeling cardiomyopathies caused by gene mutations that impact calcium transients, such as DMD [40]. Additionally, the immature nature of iPSC-CMs may lead to different electrophysiology, cell morphology, and metabolism [41,42]. 

In summary, based on our findings, we estimated that mexiletine would be effective in treating LQT3 patients with the A1656D mutation for several reasons:

- Mexiletine could reduce APD.
- Mexiletine could shift the alternant occurrence in the cell from a normal to a quicker heart rate, offering extra safety standards during treatment.
- During reentry, mexiletine could reduce the possibility of a spiral wave breakup, which can contribute to ventricular fibrillation.

## Data Availability

The data presented in this study are available on request from the corresponding author.
